# Histamine suppresses epidermal keratinocyte differentiation and impairs skin barrier function in a human skin model

**DOI:** 10.1111/all.12051

**Published:** 2012-11-15

**Authors:** M Gschwandtner, M Mildner, V Mlitz, F Gruber, L Eckhart, T Werfel, R Gutzmer, P M Elias, E Tschachler

**Affiliations:** 1 Research Division of Biology and Pathobiology of the Skin, Department of Dermatology, Medical University of ViennaVienna, Austria; 2 Division of Immunodermatology and Allergy Research, Department of Dermatology and Allergy, Hannover Medical SchoolHannover, Germany; 3 Dermatology Service, Department of Veterans Affairs Medical Center, and Department of Dermatology, UCSFSan Francisco, CA, USA; 4 CE.R.I.E.S.Neuilly, France

**Keywords:** epidermal differentiation, histamine, keratinocyte, skin barrier function, tight junction

## Abstract

**Background:**

Defects in keratinocyte differentiation and skin barrier are important features of inflammatory skin diseases like atopic dermatitis. Mast cells and their main mediator histamine are abundant in inflamed skin and thus may contribute to disease pathogenesis.

**Methods:**

Human primary keratinocytes were cultured under differentiation-promoting conditions in the presence and absence of histamine, histamine receptor agonists and antagonists. The expression of differentiation-associated genes and epidermal junction proteins was quantified by real-time PCR, Western blot, and immunofluorescence labeling. The barrier function of human skin models was tested by the application of biotin as tracer molecule.

**Results:**

The addition of histamine to human keratinocyte cultures and organotypic skin models reduced the expression of the differentiation-associated proteins keratin 1/10, filaggrin, and loricrin by 80–95%. Moreover, the addition of histamine to skin models resulted in the loss of the granular layer and thinning of the epidermis and stratum corneum by 50%. The histamine receptor H1R agonist, 2-pyridylethylamine, suppressed keratinocyte differentiation to the same extent as did histamine. Correspondingly, cetirizine, an antagonist of H1R, virtually abrogated the effect of histamine. The expression of tight junction proteins zona occludens-1, occludin, claudin-1, and claudin-4, as well as that of desmosomal junction proteins corneodesmosin and desmoglein-1, was down-regulated by histamine. The tracer molecule biotin readily penetrated the tight junction barrier of skin cultures grown in the presence of histamine, while their diffusion was completely blocked in nontreated controls.

**Conclusions:**

Our findings suggest a new mechanism by which mast cell activation and histamine release contribute to skin barrier defects in inflammatory skin diseases.

In normal skin, tightly connected keratinocytes in the stratum granulosum and terminally differentiated keratinocytes in the stratum corneum build an efficient barrier that inhibits extensive water loss while simultaneously preventing the entry of microbial pathogens and allergens into the skin (1). Dysregulation of keratinocyte differentiation together with pathologic changes in this natural barrier function is commonly associated with skin diseases such as atopic dermatitis, psoriasis, and ichthyosis vulgaris (2–4). Recently, loss-of-function mutations in the filaggrin gene have been identified (5, 6). These mutations not only impair the normal formation of the skin barrier, but also decrease the production of endogenous moisturizing molecules that lead to reduced stratum corneum hydration (4, 7). However, besides genetic defects of filaggrin production, other factors play a role in the pathogenesis of atopic dermatitis and the associated skin barrier defects as indicated by several observations: (i) The majority of individuals with atopic dermatitis do not exhibit null mutations in the filaggrin gene (8), (ii) individuals with atopic dermatitis without mutations in the filaggrin gene may also develop severe skin barrier defects (9), and (iii) a significant fraction of individuals carrying double-allele loss-of-function mutations in the filaggrin gene do not develop atopic dermatitis (4, 10).

In line with this concept, it was shown recently that the pro-inflammatory cytokines interleukin-4 (IL-4), IL-31, and TNF-α compromise barrier function (11) and modulate the expression of differentiation-associated proteins in keratinocytes (12–14). Mast cells are present in normal skin, and increased numbers of mast cells are regularly observed in the skin of patients with atopic dermatitis even before the onset of inflammation (4, 15). Mast cells release a number of important signaling molecules, among which histamine has particularly potent pro-inflammatory activities (16). After mast cell degranulation, histamine concentrations within the tissue can rise to 10–1000 μM (17), and increased histamine levels have been reported for lesional and nonlesional skin of patients with atopic dermatitis (18).

A role of endogenous histamine in the modulation of keratinocyte maturation has been suggested previously based on the observation that antihistamines have a beneficial effect on skin barrier recovery after tape striping in normal mouse skin (19). In the present study, we address the impact of histamine on the differentiation of human keratinocytes in different *in vitro* systems, among them a three-dimensional organotypic human skin model. This skin model has been shown previously to resemble native human skin, especially with regard to skin development and keratinocyte differentiation (20, 21). Our findings show that histamine prevents the expression of late differentiation antigens in keratinocytes and strongly decreases the expression of tight junction and desmosomal proteins, leading to the formation of a defective skin barrier.

## Materials and methods

### Antibodies, primers, and reagents

All the histamine receptor ligands, primers, and antibodies used are listed in Table 1.

**Table 1:** 

Histamine receptor ligands		

Receptor subtype	Agonist	Antagonist
All four receptors	Histamine*	
H1R	2-pyridylethylamine†	Cetirizine†
H2R	Amthamine†	Ranitidine†
H3R	R-α-methylhistamine†	Ciproxifan*
H4R	4-methylhistamine†	JNJ7777120†

Sequences of PCR primers		

Gene	Forward	Reverse
Alas1	5′-CCACTGGAAGAGCTGTGTGA-3′	5′-ACCCTCCAACACAACCAAAG-3′
Filaggrin	5′-AAGGTTCACATTTATTGCCAAA-3′	5′-GGATTTGCCGAAATTCCTTT-3′
Loricrin	5′-GGAGTTGGAGGTGTTTTCCA-3′	5′-ACTGGGGTTGGGAGGTAGTT-3′
Keratin 1	5′-ATCAATCTCGGTTGGATTCG-3′	5′-TCCTGCTGCAAGTTGTCAAG-3′
Keratin 5	5′-CAAGCGTACCACTGCTGAGA-3′	5′-TCAGCGATGATGCTATCCAG-3′
Keratin 10	5′-GCTGACCTGGAGATGCAAAT-3′	5′-AGCATCTTTGCGGTTTTGTT-3′
Keratin 14	5′-GACCATTGAGGACCTGAGGA-3′	5′-GGCTCTCAATCTGCATCTCC-3′

Antibodies				

			Dilution	
			
Antigen	Species	Company	Western blot	Immunostaining
Filaggrin	Mouse	Novocastra‡	1 : 200	1 : 200
Loricrin	Rabbit	Covance§	1 : 3000	1 : 1000
Keratin 10	Mouse	Abcam¶	1 : 4000	1 : 2000
Keratin 14	Mouse	Abcam¶	n.d.	1 : 200
Corneodesmosin	Sheep	R&D**	n.d.	1 : 100
Desmoglein-1	Mouse	LifeTechnologies††	1 : 200	n.d.
Zona Occludens-1	Mouse	LifeTechnologies††	1 : 250	n.d.
Occludin	Mouse	LifeTechnologies††	1 : 500	n.d.
Claudin-1	Rabbit	LifeTechnologies††	1 : 250	n.d.
Claudin-4	Rabbit	LifeTechnologies††	1 : 250	n.d.
GAPDH	Mouse	Biogenesis‡‡	1 : 2000	n.d.
Mouse IgG	Sheep	Amersham§§	1 : 10000	n.d.
Rabbit IgG	Goat	Pierce¶¶	1 : 10000	n.d.
Mouse Fluor 546	Goat	Alexa***	n.d.	1 : 500
Rabbit Fluor 546	Goat	Alexa***	n.d.	1 : 500
Sheep Fluor 546	Goat	Alexa***	n.d.	1 : 500

n.d., not done.

All PCR primers were synthesized at Microsynth (Balgach, Switzerland).

Company addresses:

* Sigma-Aldrich (Vienna, Austria);

† Tocris (Biozol, Eching, Germany);

‡ Newcastle, UK;

§ Berkeley, CA, USA;

¶ Cambridge, UK;

** Minneapolis, MN, USA;

†† Paisley, UK;

‡‡ Poole, UK;

§§ Buckinghamshire, UK;

¶¶ New York, NY, USA;

*** Eugene, OR, USA.

### Cell culture

Normal human dermal fibroblasts (Cascade Biologics, Portland, OR, USA) and normal human epidermal keratinocytes (Lonza, Basel, Switzerland) were cultured as described previously (20). Keratinocytes were cultured in 24-well plates (for the preparation of RNA) and 6-well plates (for the preparation of protein) in keratinocyte growth medium (KGM; Lonza). Keratinocytes were stimulated with histamine or histamine receptor ligands (Table 1) on the day after seeding and then every second day along with medium change until the day of harvest.

### Preparation of organotypic skin models

*In vitro* organotypic skin models were generated as previously described (20). The organotypic skin models were allowed to form a multilayered epidermis for 7 days, and the medium was changed every second day. For the stimulation of keratinocytes, histamine and histamine receptor agonists or antagonists (Table 1) were added to the skin model cultures at the time of medium change.

### *Ex vivo* culture and stimulation of skin explants

Punch biopsies (6 mm) were obtained from normal skin obtained during plastic surgery from anonymous donors directly after surgery. Biopsies were placed in keratinocyte growth medium and incubated for 3 days without or with 10 μM histamine; medium was changed every day. Thereafter, biopsies were fixed and further analyzed by hematoxylin–eosin staining and immunofluorescence labeling. The investigation of patient-derived skin biopsies was approved by the local ethics committee of the Medical University of Vienna (Vote Nr. 2011/273); the study was performed according to the Declaration of Helsinki, after patients had given their informed consent.

### RNA isolation, reverse transcription-PCR, and real-time PCR

RNA from the monolayer-cultured keratinocytes was isolated and purified using RNeasy 96 kit (Qiagen, Hilden, Germany). RNA from epidermis samples from the organotypic skin models was isolated with Trizol (Invitrogen-Life Technologies, Vienna, Austria), and RNA was purified by chloroform extraction and isopropanol precipitation. Reverse transcription-PCR was performed using iScript cDNA Synthesis Kit (Bio-Rad, Hercules, CA, USA), and real-time PCR was carried out with LightCycler 480 SYBR Green I Master (Roche Applied Science, Penzberg, Germany) according to the manufacturer's instructions. The primers used are listed in Table 1. The relative expression of the target genes was calculated by comparing with the housekeeping gene 5-aminolevulinate synthase (Alas1) using a formula described previously (22).

### SDS–PAGE and Western blot

Keratinocytes cultured in monolayer and epidermis samples from organotypic skin models were lysed in SDS–PAGE loading buffer, sonicated, centrifuged, and denatured with 0.1 M DL-dithiothreitol (DTT; Sigma-Aldrich, Vienna, Austria) before loading. SDS–PAGE was conducted on 8–18% gradient gels (GE Amersham Pharmacia Biotech, Uppsala, Switzerland). The proteins were then electrotransferred onto nitrocellulose membranes (Bio-Rad) and immunodetected with the primary and secondary antibodies listed in Table 1. Reaction products were detected by chemiluminescence with the ImmunStar™ Western C™ Substrate kit (Bio-Rad) according to the manufacturer's instructions.

### Immunofluorescence labeling

Immunofluorescence labeling of 5-μm thin sections of formalin-fixed, paraffin-embedded organotypic skin models and skin explants was performed as described previously (23). Immunofluorescence labeling and transmission light images were recorded using the AX70 microscope with the imaging software MetaMorph from Olympus (Hamburg, Germany). Merging of the sequentially acquired images and contrast adjustment was done with ImageJ 1.45s (National Institutes of Health, Bethesda, MD, USA).

### Analysis of skin permeability

Punch biopsies (6 mm) of organotypic skin models at day seven were taken and placed in a petri dish. The samples were either placed with the dermal side downwards on a 5-μl drop of LZ-Link Sulfo-NHS-LC-Biotin (biotin, 10 mg/ml; Pierce, Rockford, IL, USA) or biotin was added onto the stratum corneum of the organotypic skin models. After 60-min incubation, the samples were fixed. 5-μm sections were prepared and biotin was labeled with streptavidin conjugated to Alexa 594 (Invitrogen-Life Technologies) to visualize biotin, and the nuclei were stained with Hoechst dye (Dako). The slides were mounted and analyzed as described above.

### Statistical analysis

**Figure 1:**
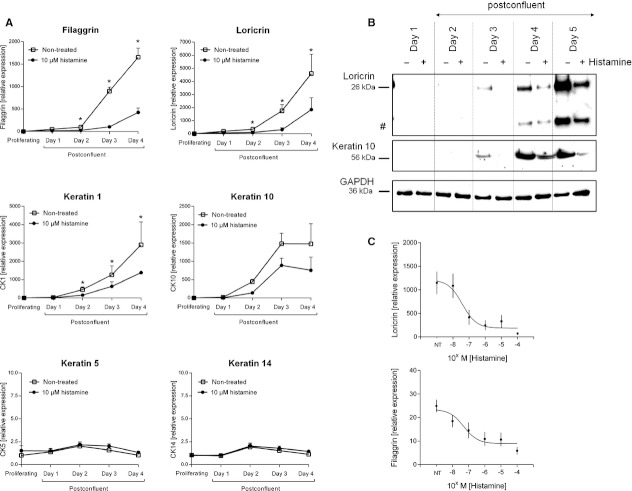
Histamine down-regulates the expression of differentiation antigens in keratinocytes cultured in monolayer. Keratinocytes were cultured postconfluent for 4 days with or without 10 μM histamine, and the expression of differentiation-associated proteins was determined by real-time PCR and Western blot at different time points of the culture. (A) mRNA expression of filaggrin, loricrin, keratin 1, and keratin 10 was down-regulated in histamine-stimulated keratinocytes, while keratin 5 and keratin 14 expression was not changed. Mean and SEM of three independent experiments each with four replicates are depicted; *P < 0.05. (B) The expression of loricrin and keratin 10 was strongly diminished at the protein level as shown by Western blot. One experiment of three is depicted. #Not identified additional band (which might be a crossreactivity with small proline-rich proteins SPRR, as the antibody used is directed against a sequence in the C-terminus of loricrin that shows high homology to SPRR). (C) Loricrin and filaggrin mRNA expression in response to different histamine concentrations in the range of 0.01–100 μM. One representative experiment of three with four replicates for each is shown.

**Figure 2:**
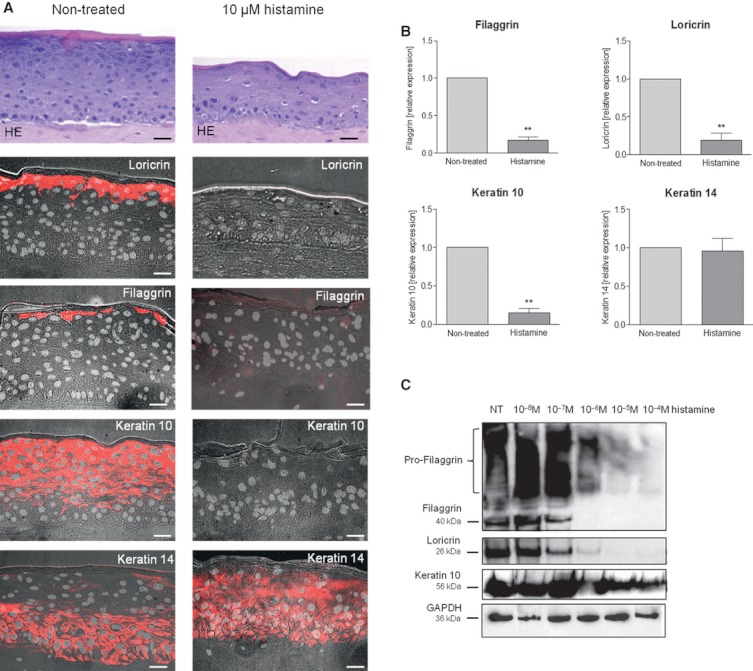
Histamine perturbs the development and differentiation of organotypic skin models. (A) After 7 days in culture, organotypic skin models were analyzed by hematoxylin–eosin (HE) staining and immunofluorescence labeling. In contrast to the controls, organotypic skin models incubated with 10 μM histamine developed a lower number of epidermal layers, lacked keratohyalin granules, and showed defects in the stratum corneum (parakeratosis). The expression of late differentiation markers loricrin, filaggrin, and keratin 10 was highly diminished in histamine-stimulated samples. One representative experiment of five is shown, bar = 20 μm. (B) Real-time PCR experiments performed with epidermal samples taken from the organotypic skin models showed a strong decrease in the mRNA expression of filaggrin, loricrin, and keratin 10 upon histamine stimulation. The expression of the basal marker keratin 14 did not change. Mean and SEM of four independent experiments each with three replicates are depicted, **P < 0.005. (C) The protein expression of filaggrin, loricrin, and keratin 10 was strongly diminished in a dose-dependent manner in histamine-stimulated skin models as shown by Western blot. One experiment of three is depicted.

Statistical analysis was performed using the program GraphPad Prism version 5 (GraphPad Software Inc., San Diego, CA, USA), and calculations are based on the means of independent experiments (each individual experiment with three to four replicates; Figs 1A, 2B, and 4A) or on the individual values of all experiments (Fig. 5A). In Figs 1A, 4A and 5A, statistical significance was calculated using analysis of variance (anova) with Bonferroni multiple comparisons post-test. In Fig. 2B, a paired *t*-test was performed. A *P*-value below 0.05 was regarded as significant and is depicted with *, and a *P*-value below 0.005 is depicted with **.

## Results

### Histamine suppresses the expression of differentiation-specific proteins in keratinocyte monolayer cultures

Human primary neonatal keratinocytes up-regulate the expression of markers of late differentiation under postconfluent culture conditions (Fig. 1; see also (24)). When histamine was added to the cultures at a concentration of 10 μM, the mRNA levels of keratin 1, keratin 10, loricrin, and filaggrin were reduced by 80–95%, whereas keratin 5 and keratin 14 mRNA levels were not altered (Fig. 1A). The corresponding protein expression for loricrin and keratin 10 was also strongly reduced as shown in Fig. 1B. The inhibitory effect of histamine on the expression of differentiation markers was dose dependent between 100 nM and 100 μM with an EC50 of 49 and 37 nM in the reduction in the expression of filaggrin and loricrin (Fig. 1C).

### Histamine impairs epidermal differentiation and stratum corneum formation in organotypic skin models

We next assessed the effect of exogenous histamine on keratinocyte differentiation in three-dimensional *in vitro* organotypic skin models (20, 23). In the absence of histamine, the epidermis of skin models comprised multiple cell layers including a distinct stratum granulosum and a stratum corneum that resemble those of native human epidermis (Fig. 2; see also (20, 23)). Exposure to 10 μM histamine during the 7-day culture perturbed epidermal differentiation and led to the lack of a stratum granulosum, a 50% reduction in the thickness of the epidermal cell layer and the stratum corneum, as well as the retention of nuclei in the stratum corneum (Fig. 2A, upper panel).

Immunofluorescence labeling showed that the differentiation markers filaggrin, loricrin, and keratin 10 that were strongly expressed in normal controls were virtually absent in histamine-exposed samples (Fig. 2A, lower panels). Keratin 14 was solely expressed in the basal layers of normal samples, but was diffusely present in all nucleated layers of the whole epidermis after histamine exposure (Fig. 2A). Both specific and dose-dependent down-regulation of these differentiation antigens was confirmed by real-time PCR and Western blotting of skin model samples (Fig. 2B,C), and by immunofluorescence labeling of the epidermis (Fig. S1). The effects of histamine were observed not only with neonatal keratinocytes, but also in skin models with keratinocytes derived from adult skin (Fig. S2). To exclude that the observed effect of histamine could be attributed to fibroblasts present in the dermal matrix of the skin models, fibroblasts were omitted in some experiments. The absence of fibroblasts did not alter the histamine-induced impairment of epidermal differentiation (Fig. S3). Furthermore, when histamine-exposed skin models were cultured for a prolonged period of time (i.e., up to 11 days instead of 7 days), epidermal differentiation remained impaired, indicating that histamine did not merely cause a delay, but rather an arrest of keratinocyte differentiation (Fig. S4).

### Histamine blocks the initiation of epidermal differentiation, but does not diminish preformed differentiation-associated proteins

**Figure 3:**
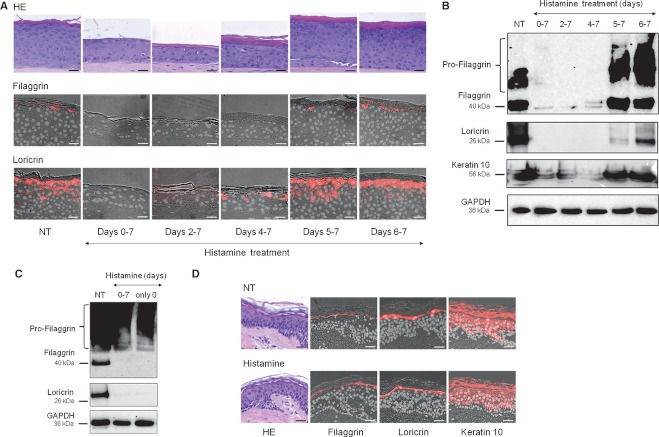
Histamine blocks the initiation of epidermal differentiation. (A, B) Organotypic skin models were incubated with histamine starting from different time points of culture. Histamine prevented the expression of differentiation-associated genes when it was added at early time points (day 1 and day 2). The addition of histamine at later time points resulted in less perturbation of differentiation as determined by HE staining and immunofluorescence labeling (A) and Western blot analysis (B). (C) When histamine was added to organotypic skin models only once at the initiation of culture (day 0), the reduction in the expression of differentiation markers as determined by Western blot analysis was comparable to that observed when histamine was added continuously for the entire culture period of 7 days. (D) When histamine was added for 3 days to explant cultures of human skin biopsies, the expression of keratinocyte differentiation markers remained unchanged. One representative experiment of three is depicted (A–D), bar = 20 μm.

The addition of histamine with every change of the culture medium (every second day), beginning at the time when skin model cultures were initiated, resulted in a pronounced defect in keratinocyte differentiation (Fig. 3A–C). The same change was observed after a single addition of histamine at the beginning of cultures (Fig. 3C), demonstrating that a continuous presence was not necessary for the action of histamine. These Western blot results were further confirmed by histology and immunofluorescence labeling (data not shown). In contrast, when histamine was added on day 4 or later, no effect on the expression of differentiation-associated proteins was observed (Fig. 3A,B).

Similarly, histamine did not modulate the abundance of late differentiation markers, when intact human skin biopsies were incubated with histamine *ex vivo* (Fig. 3D). These results suggest that histamine does not induce the degradation of preformed differentiation-associated proteins, but it rather blocks the initiation of the late differentiation program in keratinocytes.

### Histamine alters keratinocyte differentiation via activation of the histamine receptor-1

**Figure 4:**
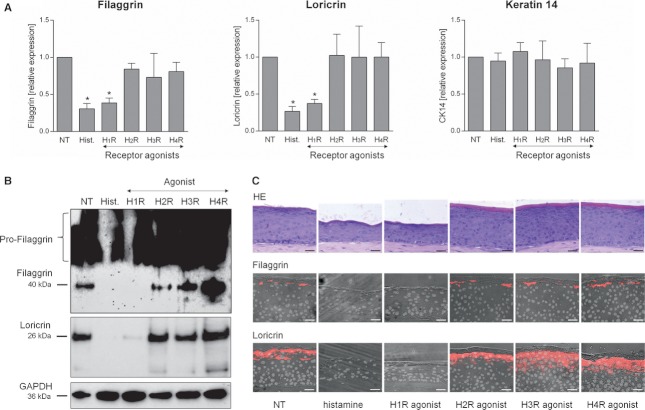
A histamine receptor-1 (H1R) agonist induces the same changes in keratinocyte differentiation as histamine. (A) Keratinocytes were cultured in monolayer, and the mRNA expression was analyzed 4 days after reaching confluence. Stimulation of keratinocytes with the H1R agonist 2-pyridylethylamine down-regulated the expression of filaggrin and loricrin; agonists of the H2R, H3R, and H4R had no effect. Mean and SEM of three (keratin 14) to four (filaggrin, loricrin) independent experiments each with four replicates are depicted, *P < 0.05. (B, C) Organotypic skin models show a deregulation of differentiation after the addition of histamine or the H1R agonist as shown by Western blot (B) as well as HE staining and immunofluorescence labeling (C). One experiment of three is depicted; bar = 20 μm.

To investigate which of the four known histamine receptors (H1R–H4R) is targeted by histamine to induce its effect on keratinocytes, selective agonists and antagonists for the different receptors were applied to keratinocytes in the presence or absence of histamine. These ligands were used at concentrations of 1–10 μM, shown in previous studies to be effective, but not toxic in keratinocytes (25, 26). The expression of differentiation proteins was inhibited by the histamine receptor-1 (H1R) agonist 2-pyridylethylamine, while agonists of the other three histamine receptors (Table 1) did not change the expression of keratinocyte differentiation markers either in monolayer cultures (Fig. 4A) or in organotypic skin models (Fig. 4B,C).

**Figure 5:**
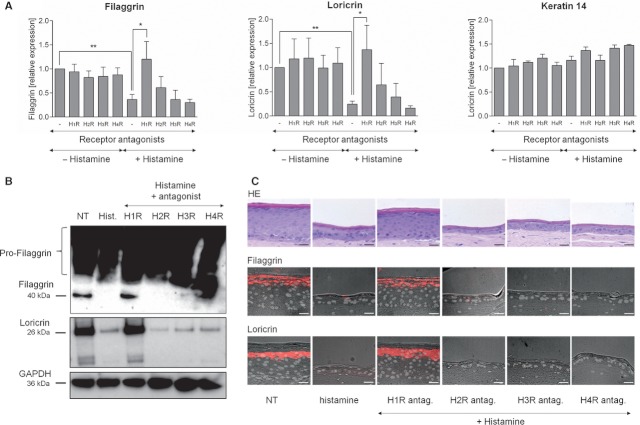
A histamine receptor-1 (H1R) antagonist blocks the effect of histamine on keratinocyte differentiation. (A) Keratinocytes were cultured in monolayer, and the mRNA expression was analyzed 4 days after reaching confluence. Incubation of the cultures with antagonists at the four histamine receptors alone did not significantly change the expression of filaggrin and loricrin. Histamine down-regulated filaggrin and loricrin expression, and if histamine was added after incubation with the antagonists, the H1R antagonist clemastine was effective in blocking the histamine effect. Mean and SEM of two independent experiments each with four replicates are depicted, *P < 0.05, **P < 0.005 (B, C) Organotypic skin models showed no histamine-induced deregulation of differentiation after preincubation with the H1R antagonist as shown by Western blot (B) as well as HE staining and immunofluorescence labeling (C). One experiment of two is depicted; bar = 20 μm.

Accordingly, preincubation of keratinocytes with the H1R antagonist, cetirizine, suppressed the histamine effect on loricrin and filaggrin expression in differentiating monolayer cultures. In contrast, the antagonists of the other three histamine receptors did not block the histamine-induced reduction in the expression of loricrin and filaggrin (Fig. 5A). Similarly, in organotypic skin models, preincubation with the H1R antagonist, but not with H2R–H4R antagonists, blocked the histamine-induced perturbation of epidermal differentiation (Fig. 5B,C).

### Histamine impairs the barrier function of the skin

**Figure 6:**
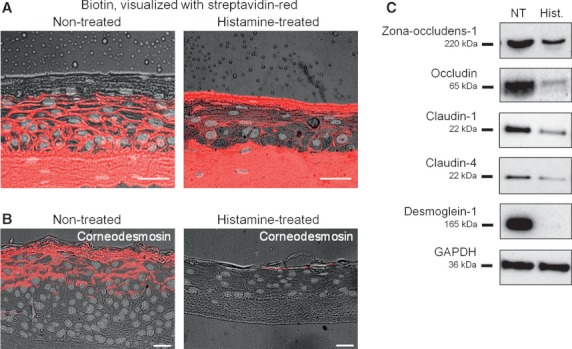
Histamine perturbs the barrier function of the skin. (A) The barrier function of organotypic skin models was investigated by applying biotin to the dermis. In control samples, biotin penetrated up to the stratum granulosum (left panel). By contrast, in the samples cultured in the presence of histamine, biotin readily penetrated through the living epidermis and accumulated in the stratum corneum (right panel). One representative experiment of three is shown; bar = 20 μm. (B) After 7 days in culture, organotypic skin models were analyzed by immunofluorescence labeling. In contrast to the control, organotypic skin models incubated with 10 μM histamine expressed no corneodesmosin. One representative experiment of five is shown, bar = 20 μm. (C) The protein expression of zona occludens-1, occludin, claudin-1, claudin-4, and desmoglein-1 was strongly diminished in histamine-stimulated organotypic skin models as shown by Western blot. One experiment of two to four is depicted.

The effect of histamine on the barrier function of the epidermis was tested by investigating the transdermal diffusion of tracer molecules. When the tracer molecule biotin was applied to the dermal side of organotypic skin models, it did not reach the stratum corneum in control samples (Fig. 6A, left panel), whereas the substance readily penetrated through all nucleated epidermal layers and accumulated in the stratum corneum of histamine-exposed samples (Fig. 6A, right panel). Similar results were obtained with the fluorescent tracer molecule Lucifer yellow (data not shown). When biotin was added to the surface of the skin model, it accumulated in the upper stratum corneum of histamine-treated and untreated samples, but did not reach the deeper epidermal layers (Fig. S5A), and the main lipid classes in the epidermis were not significantly changed by the addition of histamine (Fig. S5B). The expression of epidermal junction proteins, which normally maintain a tight skin barrier (27), was modulated in organotypic skin models in the presence of histamine: Tight junction components (occludin, zona occludens-1, claudin-1, and claudin-4) as well as desmosomal junction components (corneodesmosin and desmoglein-1) were reduced in response to histamine (Fig. 6B,C). In contrast, the adherens junction protein E-cadherin was not regulated by histamine (data not shown).

## Discussion

Atopic dermatitis is a complex and multifactorial disease, and despite the vast research efforts, its pathogenesis has not been entirely elucidated yet. It is still a matter of debate whether atopic dermatitis is triggered primarily by immunological dysregulations resulting in skin inflammation and leading to the pathological epidermal phenotype or whether the defective skin barrier is a cause rather than a consequence of inflammation. There is good evidence (8, 10) indicating that nonepithelial cells including resident and infiltrating immune cells and their mediators may play causative and important disease-modulatory roles in atopic dermatitis. Mast cells are abundantly present in lesional skin of patients with atopic dermatitis, their numbers are increased even before clinically visible skin lesions appear (15), and the histamine content is higher in the skin of patients with atopic dermatitis than in healthy individuals (18). Here, we show for the first time how histamine could aggravate atopic dermatitis via its effects on epidermal differentiation of keratinocytes. Exposure of keratinocytes to histamine resulted in reduced expression of the late differentiation markers loricrin, filaggrin, keratin 1, and keratin 10 in monolayer culture as well as in an organotypic skin model. The histamine effect was observed in a dose range of 0.1–100 μM, that is, at concentrations that are equal to or even below the concentrations measured in the skin after mast cell degranulation (17, 28).

Experiments with agonists and specific inhibitors of the four known histamine receptors (H1R–H4R; (29)) showed that histamine modulates keratinocyte differentiation via activation of H1R. Previous studies have confirmed the expression of the H1R and H2R on keratinocytes (26) and demonstrated that histamine induces changes in the expression of cytokines, cell surface molecules (e.g., MHC), and antimicrobial peptides (26, 30). Our present study demonstrates that activation of the H1R alters, in addition, the terminal differentiation program of keratinocytes.

Inflammatory mediators such as the Th2 cell–derived cytokines IL-4 and IL-31 and TNF-α have been shown to impair epidermal differentiation and have been suggested to play important roles in the pathogenesis of atopic dermatitis (12–14). Our findings on the effect of histamine extend this concept and suggest that mast cells not only mediate type I allergic reactions such as urticaria (31), but also alter epidermal homeostasis. Importantly, one single application of histamine induces this dramatic defect in terminal differentiation, indicating that a single event leading to mast cell degranulation might have a strong impact on skin barrier formation. Moreover, histamine not only delays, but rather blocks keratinocyte differentiation. Specifically, the up-regulation of multiple differentiation-associated genes was blocked when keratinocytes were exposed to histamine in their early differentiation phase, whereas the abundance of preformed differentiation markers was not reduced by the application of histamine on already differentiated *in vitro* skin (organotypic skin models) or *ex vivo* cultured native human skin.

The block of keratinocyte differentiation by histamine also resulted in a functional defect of the epidermal barrier. Histamine exposure of organotypic skin model cultures allowed the penetration of dyes from the dermis throughout the living epidermis into the stratum corneum, while in control skin the dyes could not pass the upper stratum granulosum. This difference can be explained by our observation that histamine reduced the expression of tight junction and desmosomal junction proteins. A significant reduction in the expression of the desmosomal proteins corneodesmosin and desmoglein-1 has been reported for lesional skin of patients with atopic dermatitis (31), and it was suggested that impaired tight junctions contribute to skin barrier dysfunction and immune dysregulation observed in patients with atopic dermatitis (32). These findings together with the observation that the absence of epidermal junctions leads to decreased mechanical resistance of the skin and an increased rate of water loss in mouse models (33, 34) indicate that these structural proteins are pivotal for the regulation of the skin barrier function in the upper stratum granulosum. Therefore, the impairment of tight and desmosomal junction formation by histamine might be the central mechanism leading to the observed barrier defect in the stratum granulosum of organotypic skin models. Interestingly, dye applied to the epidermal surface accumulated in the upper stratum corneum and did not penetrate into deeper epidermal layers. These results indicate that histamine does not entirely destroy the epidermal barrier, despite the suppression of the expression of several important corneocyte proteins. The main reason for this phenomenon might be the unchanged formation of the main classes of epidermal lipids in the stratum corneum of our skin models. In contrast to the situation in our organotypic skin model, the levels of different stratum corneum lipids are altered in atopic dermatitis (35). The defects in both the lipid composition and the altered expression of differentiation-associated proteins in response to histamine therefore could act synergistically and facilitate the penetration of allergens into the skin in this disease.

Urticaria that results from mast cell degranulation and massive histamine release in otherwise normal skin (36) would be a likely candidate for an impairment of the skin barrier due to histamine. However, despite intensive search, we were unable to find reports describing barrier malfunction or changes in epidermal differentiation in this disease. Our finding that histamine does not regulate the expression of differentiation markers in fully differentiated epidermis and in skin explant cultures might explain the absence of skin barrier defects in urticaria – the transitory high levels of histamine are probably not able to have a lasting effect on keratinocyte differentiation in normal skin.

Support for the hypothesis that the effect of histamine on the skin barrier is not restricted to the *in vitro* situation, but that histamine has an impact on skin barrier repair *in vivo*, comes from the observations that topical antihistamines improve skin barrier repair after tape stripping in mouse skin (19) and that barrier recovery is more efficient in mast cell–deficient mice (37). The blocking effect of histamine on keratinocyte differentiation and on the *de novo* establishment of a functional skin barrier might be of particular relevance in situations where epidermal defects and even deeper skin wounds are frequently observed, for example, in atopic dermatitis. In fact, unappeasable itch which is a cardinal feature of this disease incites compulsive scratching resulting in skin erosions. In this scenario, histamine released by allergen-induced degranulation of mast cells could affect the repair of the epidermal barrier, thus triggering a vicious cycle by facilitating the penetration of additional allergens, which then could re-induce mast cell degranulation.

In summary, our study demonstrates that histamine inhibits epidermal keratinocyte differentiation and skin barrier formation in organotypic skin models, a mechanism which might perpetuate the skin barrier defect leading to disease chronification. These findings are of potentially high relevance in skin diseases, in which skin barrier repair must proceed in the context of increased mast cell numbers and histamine concentrations such as in atopic dermatitis. Moreover, our data suggest that antihistamines, apart from their antipruritic effects, might have a positive impact on the course of atopic dermatitis, by allowing for a better repair of the skin barrier.

## Abbreviations

HxR,: histamine receptor
IL,: interleukin

## Supporting Information

**Figure S1.** The effect of histamine on epidermal differentiation is dose dependent.

**Figure S2.** Histamine perturbs differentiation in organotypic skin cultured with keratinocytes derived from adult skin.

**Figure S3.** Histamine induces the change in differentiation by acting directly on keratinocytes.

**Figure S4.** The histamine-induced changes in epidermal differentiation are still observed in organotypic skin models that were cultured for longer time period.

**Figure S5.** Biotin does not enter the epidermis when added to the surface of skin models and histamine does not modulate epidermal lipid composition.
