# Supplementary figures and images for: Histamine suppresses epidermal keratinocyte differentiation and impairs skin barrier function in a human skin model
Source: Allergy. 2012 Nov 15;68(1):37–47. doi: 10.1111/all.12051 (PMC3555427; doi:10.1111/all.12051)

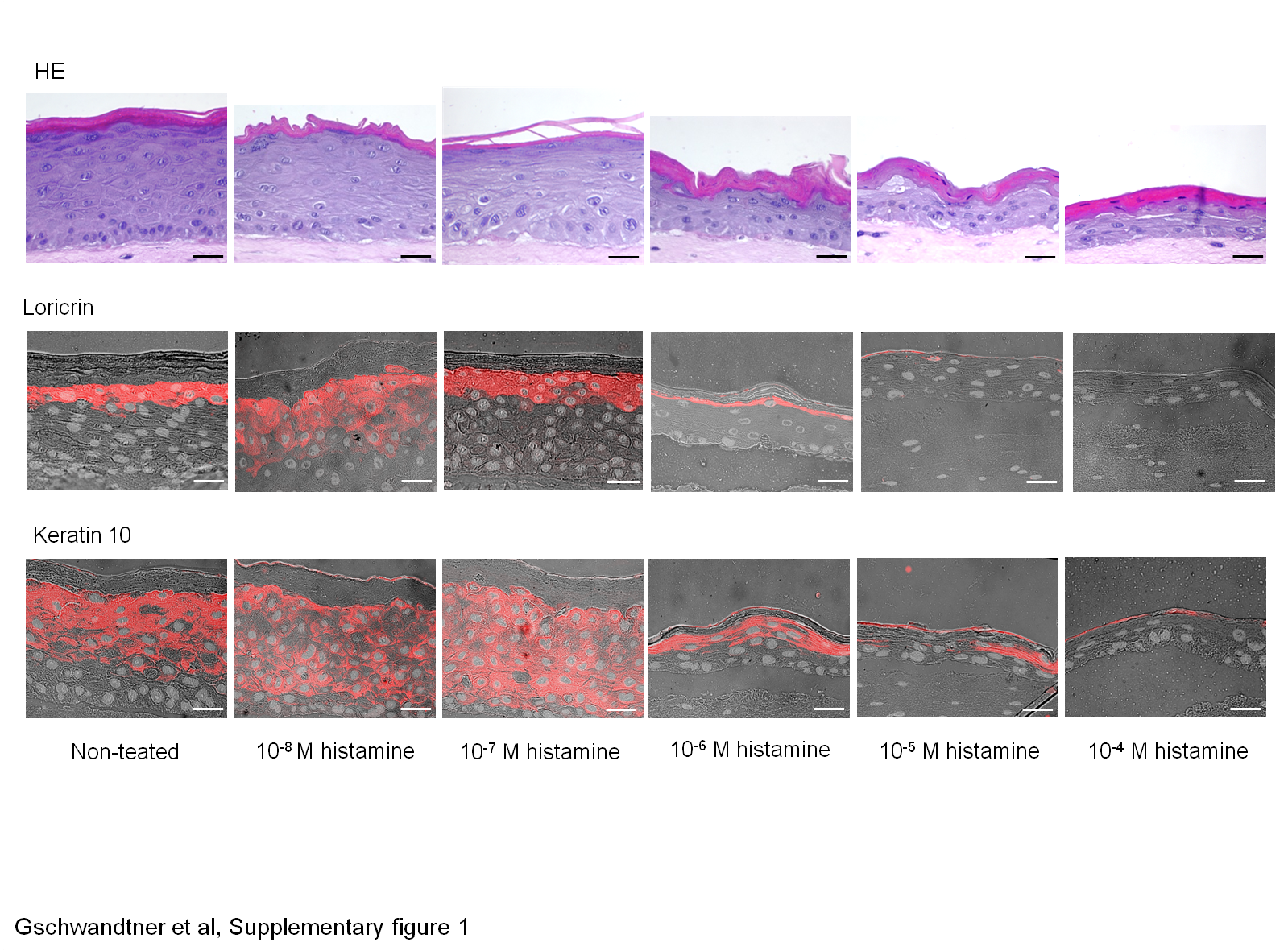

Supplement: Supplementary file 1 [file all0068-0037-SD1.tif]

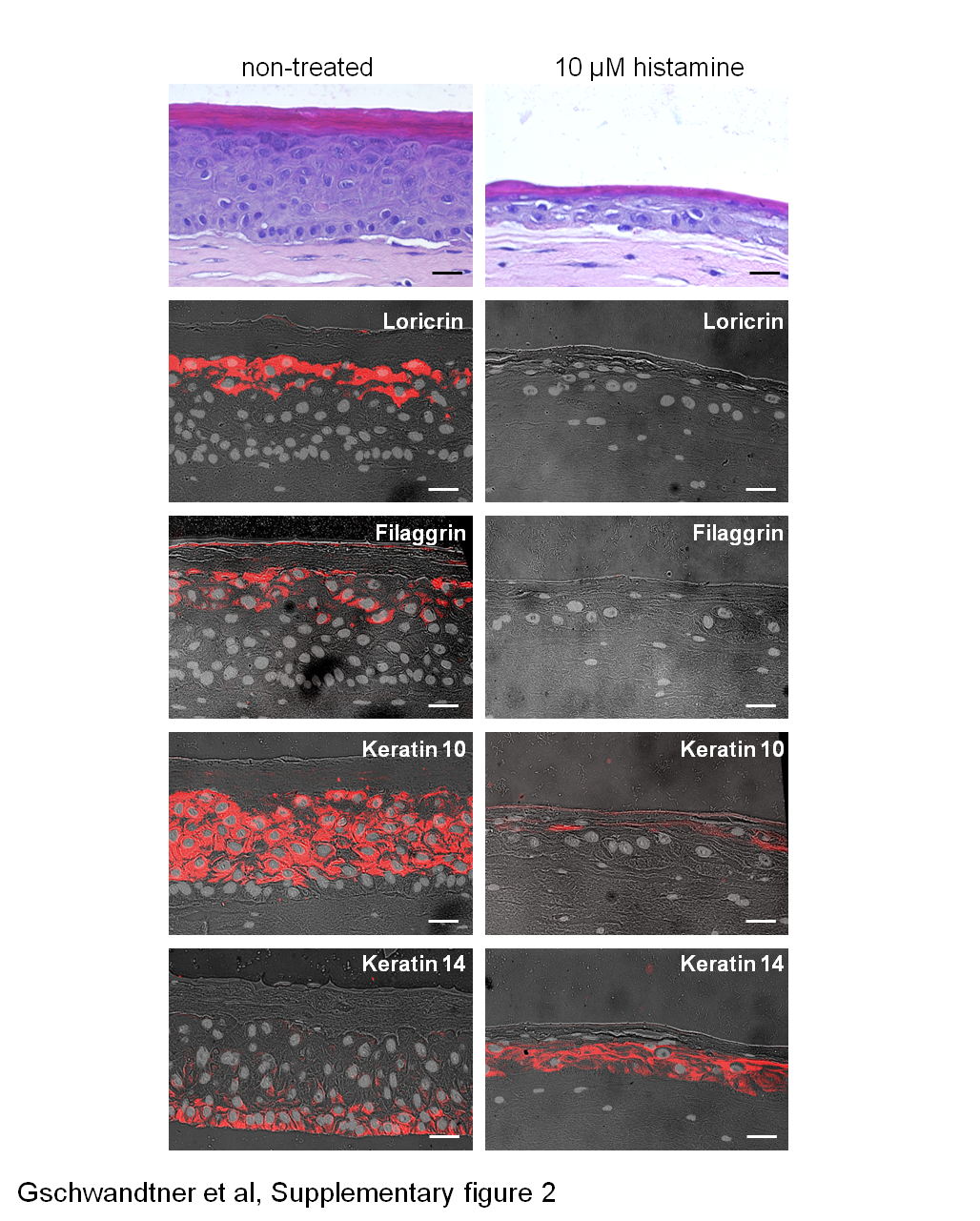

Supplement: Supplementary file 2 [file all0068-0037-SD2.tif]

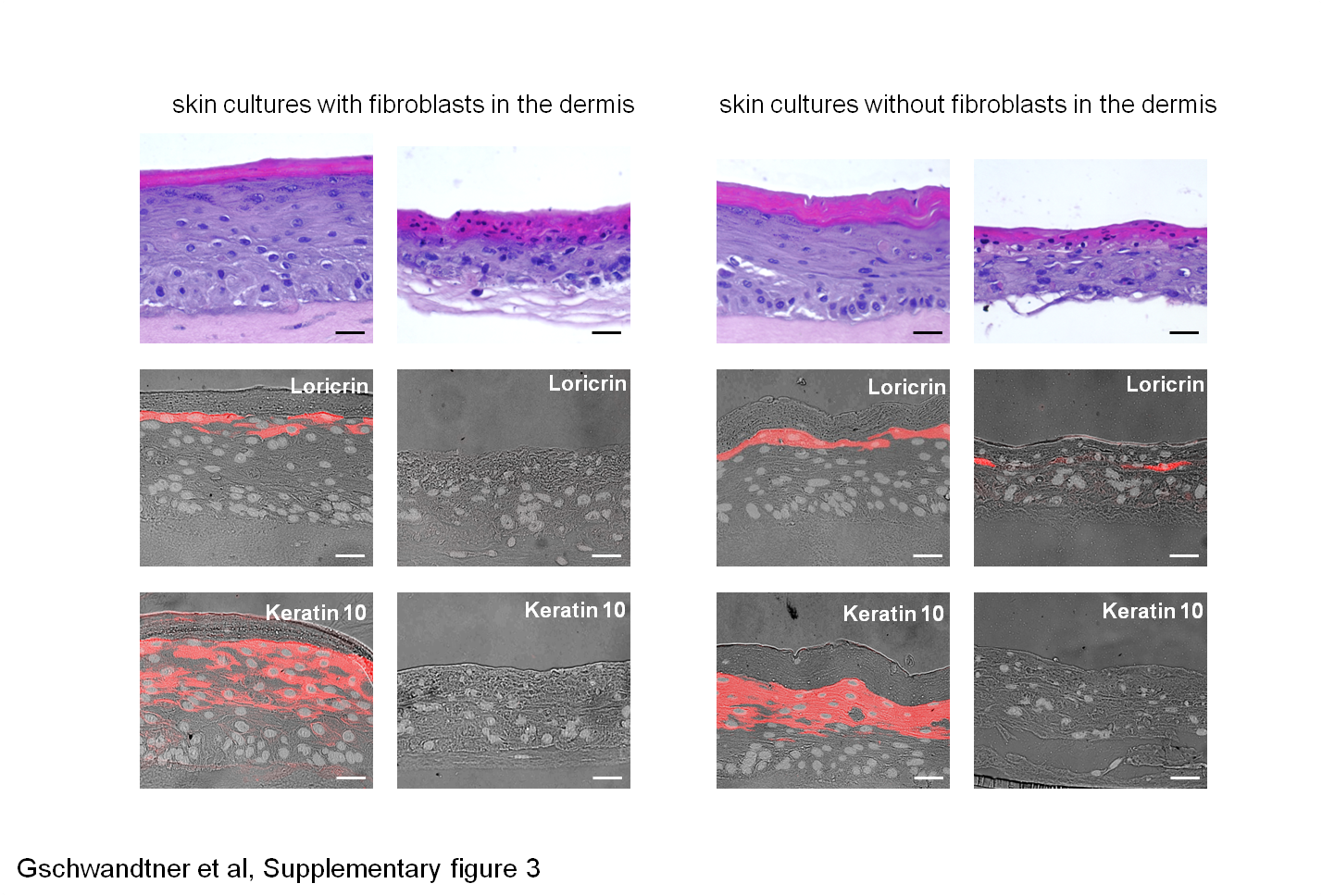

Supplement: Supplementary file 3 [file all0068-0037-SD3.tif]

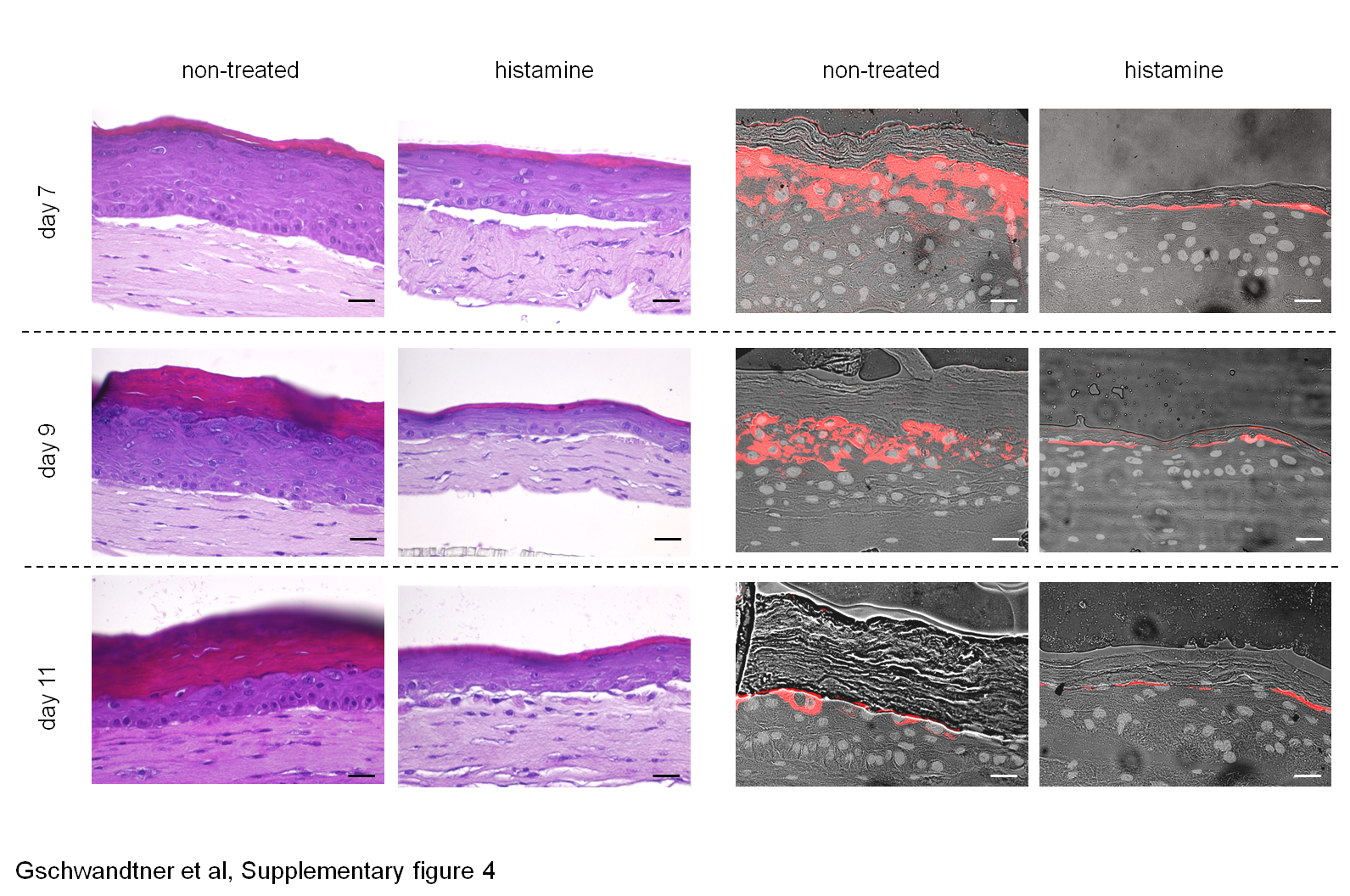

Supplement: Supplementary file 4 [file all0068-0037-SD4.tif]

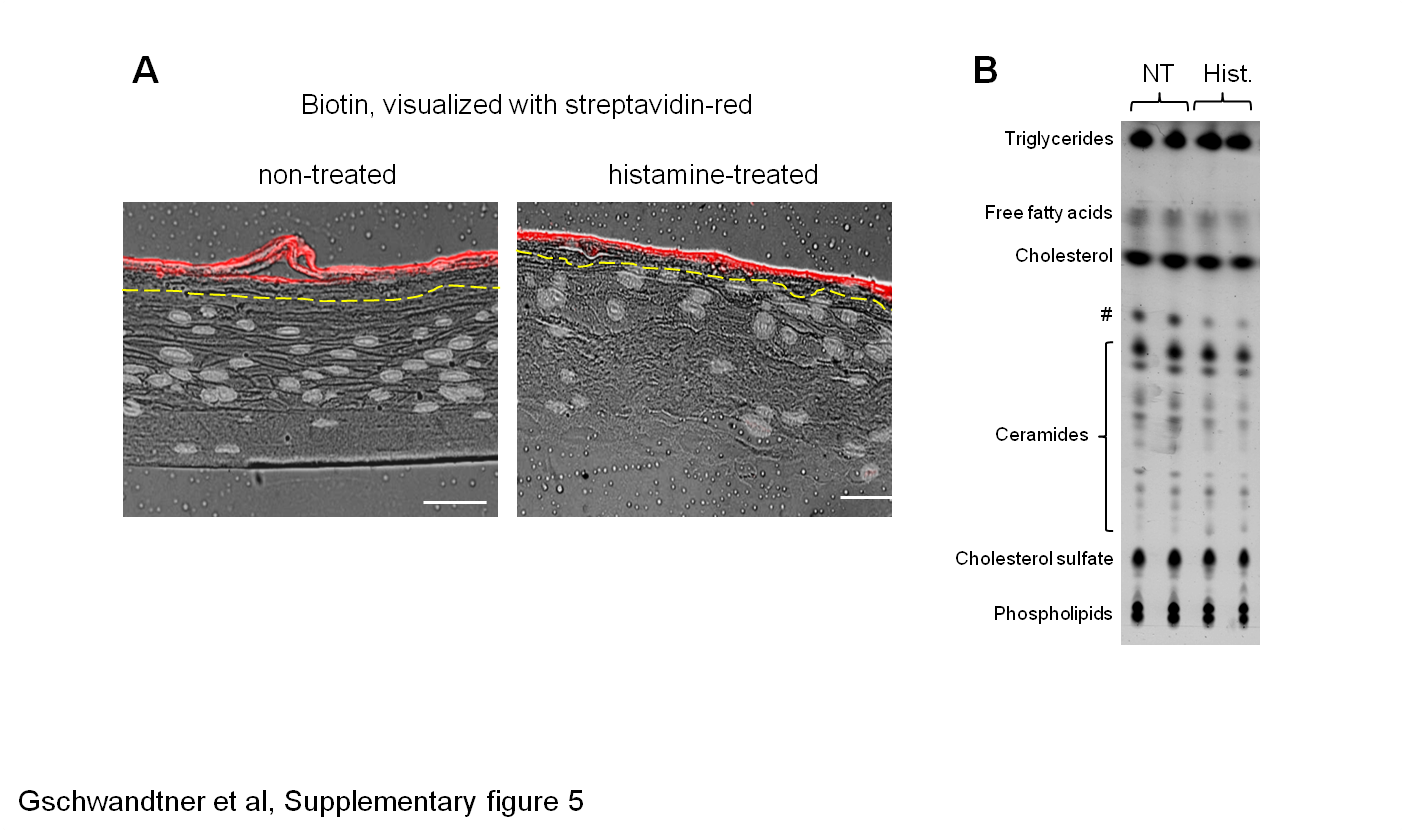

Supplement: Supplementary file 5 [file all0068-0037-SD5.tif]
