# Supplementary material for: Histamine suppresses epidermal keratinocyte differentiation and impairs skin barrier function in a human skin model
Source: Allergy. 2012 Nov 15;68(1):37–47. doi: 10.1111/all.12051 (PMC3555427; doi:10.1111/all.12051)
Supplement: Supplementary file 6 [file all0068-0037-SD6.docx]

**Supplementary figure 1:** The effect of histamine on epidermal differentiation is dose dependent. Organotypic skin models were cultured for seven days with different concentrations of histamine and analyzed by HE staining and immunofluorescence labeling. The development of a thinner epidermis, lacking keratohyalin granules and showing defects in the stratum corneum (thinning and parakeratosis) was most pronounced at histamine doses ranging from 1-100 µM and was diminished at lower concentrations. Similar observations were made for the expression of loricrin and keratin 10. One representative experiment out of three is shown; bar = 20 µm.

**Supplementary figure 2:** Histamine perturbs differentiation in organotypic skin cultured with keratinocytes derived from adult skin. Organotypic skin models were cultured for seven days and analyzed by HE staining and immunofluorescence labeling. In contrast to the controls organotypic skin models incubated with 10 µM histamine developed a lower number of epidermal layers, lacked keratohyalin granules and showed defects in the stratum corneum (thinning and parakeratosis). The expression of late differentiation markers filaggrin, loricrin and keratin 10 was highly diminished in histamine-stimulated samples. One representative experiment out of three is shown; bar = 20 µm.

**Supplementary figure 3:** Histamine induces the change in differentiation by acting directly on keratinocytes. To investigate a potential function of fibroblasts, organotypic skin models were cultured in presence or absence of fibroblasts in the dermis for seven days and analyzed by HE staining and immunofluorescence labeling. In both settings the organotypic skin models that had been incubated with 10 µM histamine developed a lower number of epidermal layers, lacked keratohyalin granules and showed defects in the stratum corneum (thinning and parakeratosis) and the expression of loricrin and keratin 10 was highly diminished. One representative experiment out of three is shown; bar = 20 µm.

**Supplementary figure 4:** The histamine-induced changes in epidermal differentiation are still observed in organotypic skin models that were cultured for longer time period. To investigate if histamine induces a delay in differentiation, organotypic skin models were cultured for longer time periods (up to eleven days), and analyzed by HE staining and immunofluorescence labeling. In long term cultured non-stimulated skin models the stratum corneum became hyperkeratotic, since natural mechanical mechanisms for its removal are missing in the *in vitro* system. In histamine-stimulated models there was no development of normal differentiated epidermis even after long term culture as observed in the HE staining and there was no significant expression of loricrin. One representative experiment out of two is shown; bar = 20 µm.

**Supplementary figure 5:** Biotin does not enter the epidermis when added to the surface of skin models and histamine does not modulate epidermal lipid composition. **(A)** Biotin was added to the surface of organotypic skin models. In control samples and histamine-stimulated samples biotin accumulated in the upper layer of the stratum corneum, but did not penetrate into the deeper stratum corneum and the living epidermis. One representative experiment of two is shown; yellow dashed line: stratum corneum-stratum granulosum boundary, bar = 20 µm; **(B)** Lipid composition in epidermis samples from skin models was analyzed by thin layer chromatography. The following solvent systems were used sequentially: chloroform/methanol/water 40:10:1 (v/v/v) to 10 cm, chloroform/methanol/acetic acid 190:9:1 (v/v/v) to 16 cm, and hexane/diethylether/acetic acid 70:30:1 (v/v/v) to 20 cm. No significant changes in the major known lipid classes were observed in presence of histamine; # represents a not yet identified lipid class.
